# Supplementary material for: Detection of AFB1 by Immunochromatographic Test Strips Based on Double-Probe Signal Amplification with Nanobody and Biotin–Streptavidin System
Source: Foods. 2024 Oct 25;13(21):3396. doi: 10.3390/foods13213396 (PMC11544864; doi:10.3390/foods13213396)
Supplement: Supplementary file 1 [file foods-13-03396-s001.zip › foods-3248535-supplementary.pdf]

Supplementary material

S1. Experimental reagents and instruments

S1.1 reagents

The recombinant vectors pET25b-G8 and *Escherichia coli BL21* (DE3) were presented by Mr Tu Chiu of Nanchang University; Dimethyl sulfoxide (DMSO) was obtained from Tianjin Zhiyuan Chemical Reagent Co. Ltd; NHS-Biotin, SA-HRP, and Maker (5-245 kDa) were purchased from Sangong Bioengineering (Shanghai) Co. Ltd; Nitrocellulose Membrane 120 was purchased from Sipo Biotechnology (Shanghai) Co. Ltd; Yeast extract and trypsin were purchased from Thermo, USA. Agar powder and TMB one-component color development solution were purchased from Beijing Solebo Technology Co. Ampicillin, IPTG and BSA were purchased from Sangong Bioengineering (Shanghai) Co. Trisodium citrate and hydroquinone were purchased from Sigma-Aldrich. Absorbent pads, gold-labeled pads, sample pads and PVC backing plates were purchased from Shanghai Jenin Biotechnology Co.

S1.2 instruments

Ultrasonic cell pulverizer (JY92-IIDN) purchased from Ningbo Xinzhi Bio-technology Co. The film scribing and spraying machine, strip cutter and chromatographic reader were purchased from Shanghai Jenin Biotechnology Co. Vacuum drying oven purchased from Shanghai Boxun Medical Biological Instrument Co. Electrophoresis apparatus was purchased from Beijing Liuyi Biotechnology Co. Constant temperature culture oscillator purchased from Shanghai Zhicheng Analytical Instrument Manufacturing Co. Autoclave sterilizer purchased from Shanghai Shen'an Medical Instrument Co. Ice machine purchased from Changshu Yiwen Refrigeration Equipment Co. The enzyme labeling instrument was purchased from Burton Instruments.

S2. Expression and purification of nanobody G8

S2.1 Prokaryotic expression of nanobody G8

A single bacterial colony was introduced into 5 mL of LB-Amp liquid medium and incubated in a constant temperature shaker set at 37 ℃ and 200 rpm for 12 hours.

Following this, the culture was transferred at a 1% inoculum volume to 100 mL of LB-Amp liquid medium, and incubated in a constant temperature shaker at 37 ℃, 220 rpm, for 3 h, so that the OD values were between 0.5 and 0.8. Subsequently, IPTG solution with a final concentration of 0.1 mmol/L was added to the cultures for induction, and the shaker temperature was changed to 25 °C at 200 rpm for 8 h to induce expression; Under the condition of 4 ℃, 10000 rpm, centrifuge the bacterial solution obtained after the above induction of expression for 15 min, discard the supernatant, accurately weigh the wet weight of the bacterium, use the bacterial cell protein lysate, carry out cell fragmentation, 4 ℃, 10000 rpm, centrifugation for 20 min, and place the supernatant at 4 ℃ for storage.

S2.2 Purification of nanobody G8

The G8 purification method is referenced and slightly modified. The column was first passed through 10 mL of ultrapure water, then the nickel column was equilibrated with 10 mL of Buffer A solution, the lysate supernatant after passing through the membrane was added, and the filtrate was caught in a centrifuge tube, then the above steps were repeated; Subsequently, non-specific adsorbed proteins were washed away with 6 mL of buffer solution with imidazole concentrations of 20 mM and 50 mM, respectively; The target proteins were then eluted with 2 mL of buffers containing imidazole at 100 mM and 200 mM. Heterogeneous proteins were subsequently washed with 1 mL of 500 mM imidazole buffer, and the nickel columns were cleaned with 8 mL of ultrapure water; Add 20% ethanol to the nickel column and place it at 4 ℃ for storage. The purification of the target proteins in the 200 mM imidazole eluate was analyzed using SDS-PAGE gel electrophoresis, and the collected target proteins were dialyzed using 0.1 M PBS buffer at 4 ℃ for 24 h. Finally, the successfully prepared nanoantibody, G8, was stored at -20 ℃.

S3. Establishment of indirect ELISA based on nanobody G8

An AFB_1-BSA solution (2 μg/mL) was incubated in 100 μL on an enzyme-labeled plate at 4 ℃ overnight. After incubation, the plate was washed three times with PBST. Next, 300 μL of 5% skimmed milk powder was added to block nonspecific binding, and the plate was incubated for 2 hours at 37 ℃. After another

three washes with PBST, nanobody G8, at concentrations of 200, 400, 800, 1600, 3200, 6400, and 12800 (100 μL each), was added to the enzyme-labeled wells containing AFB_1-BSA through serial dilution. The plates were incubated at 37 °C for 30 minutes and washed three times with PBST. After that, 100 μL of anti-His labeled secondary antibody (diluted 1:8000) was added to each well, followed by a 30-minute incubation at 37°C. After three more washes with PBST, 100 μL of TMB color development solution was added to each well and incubated at 37 °C for 5 minutes. Finally, 50 μL of stop solution was added and the OD_{450} was measured using a microplate reader.

S4. Preparation of Seed Liquid

Initially, 99 mL of ultrapure water was placed into a conical flask that had been acid-soaked overnight. Next, 1 mL of a 1% (w/w) HAuCl4 solution was added to the flask. The mixture was heated to boiling, and 2 mL of a 1% trisodium citrate solution was added rapidly while maintaining constant stirring. Stirring continued for 10 minutes during the boiling process. The solution, which turned wine red, was then cooled to room temperature and stored at 4°C.

S5. Preparation of gold nanoflowers

First, 375 μL and 750 μL of 1% $HAuCl_4$ solution were added separately to 100 mL of ultrapure water with vigorous stirring. Next, 0.5 mL of the prepared gold seeds, 220 μL of 1% trisodium citrate, and 1 mL of 30 mM hydroquinone were added sequentially to the solution. The mixture was stirred vigorously at room temperature for 30 minutes. The colloidal gold particles were then analyzed using full-band UV scanning within the wavelength range of 400-800 nm and characterized by TEM to observe their morphology and measure their size.

S6. Optimization of test strip parameters

S6.1 Optimal pH optimization of AuNFs@ G8-Bio probes

Add 1 mL of small particle size AuNFs solution in a beaker, adjust the pH to 5.5, 6.0, 6.5, and 7.0 with 1% K_2CO_3 solution, add an appropriate amount of G8-Biotin nanoantibody to each beaker for labeling, centrifugation, and resuspend the precipitated gold-labeled probes with gold-labeled probe resuspension solution, and

record the OD_T value with a chromatographic readout instrument by observing the color of the T and C lines, and determine the optimum pH based on the average OD_T of the three experiments for each experiment for three parallel times. The optimal pH was determined based on the average OD_T values of the three experiments.

S6.2 Optimization of the amount of G8-Biotin nanobody

The appropriate amount of antibody is crucial for the detection sensitivity of the test strips. A series of volumes of G8-Biotin nanoantibodies (10 μL, 15 μL, 20 μL, and 25 μL) were added to a solution of small particle-size AuNFs at the optimal pH to prepare AuNFs@G8-Bio probes, and the optimal nanoantibody dosage was determined by recording OD_T values through the chromatographic readout instrument.

S6.3 Optimization of pH for AuNFs@SA probes

1 mL of large particle size AuNFs solution was added to a beaker, and the pH was adjusted to 5.5, 6.0, 6.5, and 7.0 using a 1% K_2CO_3 solution. 5 μL of SA solution was added to each beaker for labeling. The mixture was centrifuged, and the precipitated gold-labeled probes were resuspended in gold-labeled probe resuspension solution. The optimal pH was determined by observing the color of the T and C lines, and the OD_T value was recorded with the chromatographic readout apparatus.

S6.4 Optimization of SA concentration

The AuNFs@SA probe was prepared by adding 5 μL of a series of concentrations of SA solution (0.2 mg/mL, 0.4 mg/mL, 0.6 mg/mL, and 0.8 mg/mL) to a solution of large-size AuNFs at the optimal pH, and the optimal concentration of SA was determined by observing the color of the T and C lines, and recording the OD_T value with a chromatography reader.

S6.5 Optimization of AuNFs@G8-Bio and AuNFs@SA Position and Scaling

The position of the expansion pad can directly affect the effect of signal amplification. Separately place the gold standard pad and the expansion pad on top, drop the probe and observe the color of T and C lines. Set up a series of dosage ratios of AuNFs@G8-Bio and AuNFs@SA (2 μL AuNFs@G8-Bio, 2 μL AuNFs@SA, 2:1, 1:1, 2:3, 1:2), observe the T and C line colors, and record the OD_T values with a

chromatographic readout to determine the optimal ratio.

S6.6 Optimization of T and C line concentrations

The AFB_1-BSA artificial antigen was diluted to 300 μg/mL, 250 μg/mL, 200 μg/mL, and 150 μg/mL with PBS, and encapsulated on NC membrane as T-line using a membrane scribing and gold spraying all-in-one machine at 0.8 μL/cm, and the OD_T value was recorded with a chromatographic readout instrument. Bio-BSA was diluted to 0.2 mg/mL, 0.3 mg/mL, 0.4 mg/mL, and 0.5 mg/mL with PBS, and then encapsulated it on NC membrane as C line using a gold-spraying film scribe at a speed of 0.8 μL/cm, observed the colors of T and C lines, and recorded the OD_T/OD_C values with a chromatographic readout to determine the optimal T and C line concentrations.

S7. Introduction to the standard curve for immunochromatographic test strips

B/B_0 as the vertical coordinate (B represents the OD_T/OD_C value of the test strip used to detect the series of standards, while B_0 represents the OD_T/OD_C value of the test strip used to detect the negative control samples), the logarithmic value of the concentration of AFB_1 solution as the horizontal coordinate, the standard curve of the test strips for the quantitative detection of AFB_1 was plotted, and the IC_{50} and LOD of the test strips were calculated, with the IC_{50} being the concentration of AFB_1 corresponding to an inhibition of 50%, and the LOD being the concentration of AFB_1 corresponding to an inhibition of 10%.

S8.Treatment of maize samples

The corn samples were ground into powder and passed through a 20-mesh sieve. Five grams of the powdered corn samples were accurately weighed, and 1 mL of an AFB_1 standard solution was added. The samples were dried at room temperature, followed by the addition of 10 mL of extraction reagent (methanol: water, 7:3). The mixture was placed in an oscillator and agitated for 30 minutes. Afterward, it was centrifuged at 5000 r/min for 10 minutes, and the supernatant was collected. The supernatant was then filtered through a 0.22 μm filter and subsequently diluted tenfold with buffer solution to achieve final AFB_1 concentrations of 0, 0.1, 1, and 10 ng/mL.
